# “We Need to Learn from What we Have Learned!”: The Possible Impact of Covid-19 on the Education and Training of Chaplains 

**DOI:** 10.1177/1542305021996228

**Published:** 2021-04

**Authors:** Eleanor Flynn, Heather Tan, Anne Vandenhoeck

**Affiliations:** University of Divinity, Melbourne, Australia; Spiritual Health Association, Melbourne, Australia; KU University, Leuven, Belgium

**Keywords:** Education, spiritual care, COVID-19, chaplaincy

## Abstract

The responses of chaplains providing care in health services during the Covid-19 pandemic showed that they both learned new skills and taught these to others while working in environments made unfamiliar by personal protective equipment and social distancing. This paper discusses the responses of the participants as they relate to education and training as well as suggesting new content and styles of education to meet the needs of chaplains in future similar events.

## Introduction

The education of chaplains who work in the health sector varies across the globe and between organisations within countries and regions with some being theologically based while others are more health based and some combine elements of both (Cadge et al., 2019). While some education programs include caring for people in crisis situations (Martens, 2004), it is unlikely that many of the participants in the recent international survey of chaplains and spiritual care workers would have received such training.^2^ While the overwhelming majority of the respondents had relevant postgraduate qualifications it is not likely that their training will have covered providing spiritual care in a pandemic situation nor how to best use the many technologies being rolled out to assist everyone communicate in this situation. Another important aspect of education in spiritual care is the provision of ongoing supervision and support, which the great majority of the participants in this survey were receiving, albeit sometimes through new technologies.

From our review of the survey data we consider that the following issues are those that chaplains needed to obtain new knowledge about or skills in so they could care for patients, families and staff in the pandemic and therefore those that merit the development of future education programs:

1. using new technologies to support patients, families and other health staff, including developing information on how to access spiritual care,
2. working in a crisis, including the need for protective equipment,
3. providing spiritual care for people of other faiths,
4. working with other disciplines to enable them to provide spiritual support,
5. supervision and continuing education using new technologies
6. advocating for the role of chaplains in healthcare, including in crises

## Results

The survey responses show that while only a minority of Australian respondents provided care for patients’ family members this was more common in Europe and a majority in North America. Many mentioned providing care to families via new technologies at critical times, and the constraints involved

*Having to conduct long, intense conversations by phone…. I never met in person* [P311 male 42 Europe]

The lack of physical presence, non-verbal expressions and touch was also mentioned several times

*Virtual sessions are not the same as in-person sessions. I prefer video sessions over phone sessions as I like to at least be able to see the client. It is more challenging to read body language and gestures by video as I cannot see the client's whole body…usually just their face and shoulders. However, it is amazing to experience that I am still able to build rapport, join in a therapeutic alliance and share meaningful moments of connection during video sessions.* [P1646 female 50 Nth. America]

We found that the great majority of respondents across all continents provided spiritual care and support to other staff often or most of the time

*offering meditation for staff and debriefing* [P309 male 60 Australia],

as well as providing regular emails and phone calls or newsletters for staff wellbeing.

Some commented on changes in their workload or roles

*For me it was new to support the Medical Staff and Crisis Team the whole time….During the pandemic we had to make a protocol for allocation on the intensive care. And I supported medical practitioners* [P468 female 57 Europe].

while for others it was not obvious how much of a change from their usual roles the provision of care for family members and staff was, nor how prepared they felt for these roles. We discovered that staff had to train themselves and then family members in using technology to interact with patients in isolation.

*We did a LOT of phone calls to families and helped to get them connected to the patient and to the team.* [P360 female 42 Nth. America].

Chaplains also commented they regularly used video conferencing technology to participate in clinical team and management meetings. Many of those surveyed were not solely providing support for patients, families or staff but were involved in all aspects of patient care

*discussing triage scenarios in ethical context, connecting people via tablet, phoning with patients' relatives more often, take-away sermons and services* [P60 female 44 Europe]

However, some only provided care to patients

*Spiritual care is to walk the walk, not talk the talk. ….We did not engage in discussions, meetings, clinical lessons, peer support, etc etc ….* [P151 female 62 Europe].

Interestingly nearly one third of the respondents, 538, from all regions reported that spiritual care was provided by other staff during the Covid-19 pandemic, sometimes because volunteers were not allowed to visit or because the whole department had been sent home

*preparing lots of resources for staff to use in the absence of a chaplain/faith rep* [P278 male 55 Europe]

although at some sites when other healthcare staff offered to assist with the provision of spiritual care for areas where chaplains could not visit this was not always well received

*Some specialist nursing staff felt they could move into the chaplaincy department and provide chaplaincy support. However, this was not necessary, it was respectfully pointed out to them the education and training needed to work in this professional environment.* [P1591 female 62 Europe]

Another new aspect of care for chaplains who were asked to provide rituals for people of other faiths

*We had a huge learning curve with our first Muslim patient. How to honour their ritual and traditions vs pandemic safety.* [P29 female 28 Nth. America]

The degree to which senior chaplains were included at management level planning of the Covid-19 response varied greatly from a role that raised the profile of chaplaincy to less positive ones

*We were essential from the planning stages, and our role grew with more patients that we received.* [P107 female 57 Nth. America]

*Chaplain leadership should have been at the executive leadership, how to address the pandemic.* [P41 male 54 Nth. America]

This raises the issue of advocacy and the degree to which chaplains can speak for spiritual care and its role in whole person care in interdisciplinary settings.

*Having the opportunity to say what I could offer and answer staff questions if required (would have been good)* [P759 male 58 Europe].

The impacts of Covid-19 on the education and training of chaplains varied across the regions surveyed. A majority of chaplains in all continents, 1157 out of 1657, were members of a professional association. The frequency of supervision during the pandemic remained reasonably high across all continents. Professional associations offered more professional and educational support to chaplains than faith organizations, as expected given it is their core mission. North American based professional organizations offered more online webinars and opportunities to share professional knowledge than Australian with European ones offering the least.

## Discussion

In considering the future educational needs of chaplains both the content and style of the education needs to change because chaplains have not been previously educated or prepared for such an extended pandemic. (Karle, 2020)

*As chaplains, we have much to learn about dealing with pandemic events (or any major crisis of mass proportions), & it seems we are really not provided more than basic tools to consider/prepare to deal with these.* [P293 male 75 M Nth. America]

1. Educational programs should include capacities based on the learnings from other crises such as explosions, fires, major accidents and war. The specific crisis capacities are: calming people in the crisis, using information to provide structure for them, discovering resources and then making them available, facilitating other professionals to provide spiritual care, performing rituals in unusual situations, providing staff care and support, integrating elements of spiritual care into the team, appropriately referring people for specialized help, and connecting people’s experience with meaning. (Martens, 2004)
2. Chaplains will need to be taught explicitly how to use the various technologies for interacting at a distance with patients, family members and other staff and how to use personal protective equipment correctly. As more than 70 percent of respondents agreed that they would retain aspects of their online ministries when restrictions on public gatherings are lifted, it is necessary for educators and trainers to discern what kind of training is needed in order to provide the best possible spiritual care online (Sprik et al., 2020). Communication skills will need to be taught and practised in simulation sessions (Carrad et al., 2020) so that everyone understands how to see and hear clearly, but more importantly how to listen and watch carefully so the nuances of people’s conversations and emotions are picked up and can be discussed and supported. As the participants told us many of the skills can be picked up relatively quickly

*tele-chaplaincy and virtual face-time chaplaincy. But all learned quickly and adjusted well* [P1483 male 50 Nth. America]

However, it is important that the skills are reviewed so that any poor practices are not repeated because of lack of proper educational input at the time the skills are learned.

3. Because health care facilities limit outsider visits during crises chaplains will need to attend to the spiritual needs of people of different faiths to their own or those with none, sometimes leading rituals normally offered by other faith practitioners. So, it will be necessary to teach the principles of general spiritual care, knowledge of other faith rituals, and working with other disciplines while enabling chaplains to communicate their own specialist spiritual care (Vandenhoeck, 2021). Education programs are being developed for other health care staff to identify spiritual needs in patients and make appropriate referrals to the professional chaplaincy team (Puchalski et al., 2019). Indeed, some models assume that some spiritual care is provided by many healthcare staff with the specialist chaplains attending only to specific referrals and more complex situations (Austin et al., 2017; Gordon & Mitchell, 2004; Tan et al., 2020).
4. The Covid-19 pandemic has put attending live classes, conferences and other forms of professional development on hold. Online alternatives such as webinars, sharing circles, supervision have become more prominent. Educational institutions are working to provide models of education and interaction that will not replicate the live conference or seminar but will engage the participants and ensure chaplains have the necessary tools to provide spiritual care at all times, including in major crises. *We need a centralized repository of online resources.* [P906 female 46 Nth. America]
5. Other contributions in this issue have already pointed out that there were great differences in whether chaplains were involved in providing care during the pandemic. It seems therefore of utmost importance that educational organizations and professional associations educate chaplains to advocate for their role in caring for patients and staff. There is a need to educate both chaplains and the higher level management of healthcare systems, about the need for a professional work force (Best et al., 2020; Kim et al., 2020). As one participant expressed it:

*Management and the chaplaincy bodies need to do more in elevating the role of the chaplain. Management need to understand about the value of chaplains even though we do not have a financial bottom line. Also, many managers were not proactive and this almost caused the death of our department professional bodies need to train members how to make a difference and to bring value to the profession.* [P1184 female 44 Nth. America]

## Conclusion

The participant quoted below expresses many of our thoughts on the need for education in and about the pandemic, because all patients (not just those with Covid) must be provided with appropriate and timely spiritual care by chaplains who are educated in safe clinical practice and best use of technology to provide care to all patients, family members and staff.

“*Depending on your local experience, the learning and outcomes of this experience will continue to be revealed and reflected upon for a long time to come. The challenge is to now integrate the care of the patient with COVID as normal practice and advocate for their needs and autonomy. We are moving out of the crisis phase of management so now all the other really important principles of safe and quality care need to be reassessed in the light of living with this virus into the foreseeable future*.” [P 413 female 63 Australia]

## Notes

1 The quote in the title comes from closing comment (P643 North America female 72) “This is a great idea. We need to learn from what we have learned! …”

2 For the aims, ethics approval, survey questions and analysis of the quantitative questions, we refer to the article of Austyn Snowden in this special issue.
